# Evaluating the Association Between Methylenetetrahydrofolate Reductase (Rs1801131 and Rs1801133) Gene Polymorphisms and Severity of Coronary Lesions in Patients With STEMI and NSTEMI: A Retrospective Cross‐Sectional Study

**DOI:** 10.1002/hsr2.70284

**Published:** 2025-01-12

**Authors:** Behnam Nazarzadeh, Saeedeh sadat Ghazanfari, Farzaneh Karimi, Seyed ali Moezibady, Fatemeh Salmani, Kazem Dastjerdi, Hamidreza Mohammadi

**Affiliations:** ^1^ Department of Medical Biotechnology, Faculty of Medicine Birjand University of Medical Sciences Birjand Iran; ^2^ Mashhad University of Medical Sciences Mashhad Branch Islamic Azad University Mashhad Iran; ^3^ Institute of Medical Biochemistry and Molecular Biology, University Medicine Greifswald University of Greifswald Greifswald Germany; ^4^ Department of Biology, Faculty of Sciences Ferdowsi University of Mashhad Mashhad Iran; ^5^ Razi Clinical Research Development Unit (RCRDU) Birjand University of Medical Sciences Birjand Iran; ^6^ Social Determinants of Health Research Center, Department of Epidemiology and Biostatistics, School of Health Birjand University of Medical Sciences Birjand Iran; ^7^ Cellular and Molecular Research Center Birjand University of Medical Sciences Birjand Iran; ^8^ Yazd Cardiovascular Research Center, Non‐communicable Diseases Research Institute Shahid Sadoughi University of Medical Sciences Yazd Iran

**Keywords:** coronary artery disease, MTHFR, Rs1801131, Rs1801133

## Abstract

**Background and Aims:**

Mounting evidence have implicated that rs1801131 and rs1801133, located in the Methylenetetrahydrofolate reductase (MTHFR) gene, may emerge as novel biomarkers for coronary artery disease (CAD). The Synergy between Percutaneous Coronary Intervention with Taxus and Cardiac Surgery (SYNTAX) score is also an appropriate predictor for revascularization strategy in patients with complex CAD. The aim of this study is to investigate the correlation between rs1801131 and rs1801133 with the severity of coronary lesions in patients with ST‑Elevation Myocardial Infarction (STEMI) and Non‑ST‑Elevation Myocardial Infarction (NSTEMI) based on the SYNTAX score.

**Methods:**

This retrospective cross‐sectional study included 96 patients diagnosed with STEMI and NSTEMI from Razi University Hospital between April and September 2019. Ninety‐six patients were diagnosed with STEMI (*N* = 43) and NSTEMI (*N* = 53) were recruited from South Khorasan, Iran. The angiographical characteristics of CAD were defined by the SYNTAX score. Genomic DNA was isolated from peripheral blood and genotyped for rs1801131 and rs1801133 using the TaqMan real‐time PCR method.

**Results:**

The results of the one‐way analysis of variance indicated that there is no association between rs1801131 and rs1801133 with the severity of coronary lesions in patients with STEMI (*p* = 0.44) and NSTEMI (*p* = 0.91). However, the two‐way analysis of variance comparison and post‐hoc test demonstrated that rs1801133 in the presence of rs1801131 is correlated with the SYNTAX score in NSTEMI (*p* = 0.03) and total patients (*p* = 0.03).

**Conclusion:**

In conclusion, our study reveals a significant association between the MTHFR polymorphism rs1801133 and CAD severity, particularly in NSTEMI patients. While rs1801131 showed no correlation, rs1801133 may serve as a valuable genetic biomarker for assessing CAD severity. Further research with larger populations is needed to confirm these findings.

**Abbreviations**
ApoA‐I: apolipoprotein A‐I
CABG: coronary artery bypass grafting
CAD: coronary artery disease
CVA: cerebrovascular accident
HLP: hyperlipidemia
HTN: hypertension
IHD: ischemic heart disease
MTHFR: methylenetetrahydrofolate reductase
NSTEMI: non‑ST‑elevation myocardial infarction
PCI: percutaneous coronary intervention
PTE: pulmonary thromboembolism
STEMI: ST‑elevation myocardial infarction
SYNTAX: Synergy between Percutaneous Coronary Intervention with Taxus and Cardiac Surgery
WHO: World Health Organization

## 1 Introduction

Based on the World Health Organization (WHO) reports, cardiovascular disease is the most common cause of mortality worldwide. Approximately 17.9 million people diagnosed with cardiovascular disease died in 2015, while 7.3 million of them were coronary artery disease (CAD). Over recent years, several studies have revealed that genetic factors play a critical role in susceptibility to cardiovascular disease, particularly CAD [1]. The anatomical Synergy between percutaneous coronary intervention (PCI) with Taxus and Cardiac Surgery (SYNTAX) score is a comprehensive angiographic scoring system, which objectively guides the decision‐making process between coronary artery bypass grafting (CABG) surgery and PCI [2]. The clinical outcomes are comparable with PCI and CABG in patients with low (0–22) and intermediate (23–32) SYNTAX scores, while clinical outcomes are better with CABG in patients with a high score (≥ 33) [3]. SYNTAX score model is considered a practical tool to choose the appropriate revascularization treatment in patients with complex CAD [4].

Demethylation of methionine leads to produce homocysteine, a sulfur‐containing essential amino acid. This amino acid contributes to oxidative stress, platelet activation, endothelial dysfunction, and thrombus formation, which induce vascular lesions [5, 6]. Emerging evidence has shown that homocysteine is considered a prominent risk factor for cardiovascular disease even in young patients [5, 7]. Furthermore, it has been observed that higher levels of homocysteine serum are correlated with the severity of CAD [8]. The metabolism of homocysteine consists of remethylation and transsulfuration pathways. During the remethylation, the blood folate provides a methyl group to homocysteine, forming tetrahydrofolate, which finally is converted into methionine. The transsulfuration pathway includes homocysteine reactions with serine, converted into cystathionine, cysteine, and then sulfates [6, 9]. Methylenetetrahydrofolate reductase (*MTHFR*) is a critical enzyme in methionine metabolism. It is a co‐substrate in the homocysteine remethylation pathway, which can convert homocysteine to methionine through catalyzing an irreversible conversion of 5,10‐methylenetetrahydrofolate to 5‐methyltetrahydrofolate and providing methyl [5]. Numerous mutations in *MTHFR* lead to inappropriate activity of *MTHFR*. Several diseases are found to be correlated with *MTHFR* polymorphisms including cardiovascular disease, Alzheimer's, neural tube failure, and brain diseases [10]. Rs1801131 and rs1801133 are common variants of the *MTHFR* gene [11, 12]. Several studies have suggested that these two SNPs can enhance the risk of CAD [13, 29], but the correlation between these SNPs and the severity of CAD has been poorly studied [14]. Although several studies have examined the role of MTHFR polymorphisms in cardiovascular disease, particularly CAD, the association between these polymorphisms and the severity of CAD remains poorly understood. The existing research is limited in terms of sample size, ethnic diversity, and consistent findings across different studies. Furthermore, few studies have explored the role of these polymorphisms in specific subgroups, such as patients with ST‐elevation myocardial infarction (STEMI) and non‐ST‐elevation myocardial infarction (NSTEMI). Thus, we conducted this study to investigate the hypothesis that there is an association between *MTHFR* polymorphisms (rs1801131 and rs1801133) and the severity of coronary lesions in patients with STEMI and NSTEMI.

## 2 Materials and Methods

### 2.1 Study Population

This retrospective cross‐sectional study included 96 patients diagnosed with STEMI and NSTEMI from Razi University Hospital between April and September 2019. Coronary angiography was conducted by an experienced cardiologist. The severity of CAD was also assessed according to the SYNTAX scores. The present study was approved by the ethics committee for the human subject study of Birjand University of Medical Sciences (Ethic code: IR.BUMS.REC.1397.351). The ethical principles described by the guidelines of the Helsinki Declaration were exerted in Human Research. All participants were given written informed consent. Patients with congenital heart disease, diabetes, renal failure, severe infection, hepatic failure, tumor malignancy, and history of B6, B12, and folic acid intake were excluded. The demographic characteristics including age and sex, were recorded. Additionally, the medical history of hyperlipidemia (HLP), hypertension (HTN), renal stone, Hypothyroidism, ischemic heart disease (IHD), cerebrovascular accident (CVA), and pulmonary thromboembolism (PTE) were recorded.

### 2.2 DNA Extraction and Genotyping

Genomic DNA was isolated from 200 µL EDTA anticoagulant peripheral blood samples from all participants using a QIAamp Blood Mini Kit (USA), according to the manufacturer's protocol. The purity and the concentration of extracted DNA were quantified by a spectrophotometer (Thermo Scientific, USA) and stored at –20°C for further analysis. Genotyping of the rs1801131 and rs1801133 was performed using TaqMan probe real‐time PCR assay with MTHFR C677T and MTHFR A1298C kits (Nic Gene O Gene Biotech Company, Iran) including NicGOG‐MTHFR c677t Genotyping Real‐time PCR kits and NicGOG‐MTHFR a1298c Genotyping Real‐time PCR kits based on the kits manual and carried out on the Rotor‐Gene Q MDx 5plex HRM instrument. The reaction mixture was subjected to 50 cycles of 15 s denaturation at 60°C and 60 s annealing at 61°C.

### 2.3 Assessment of SYNTAX Score

Calculation of the SYNTAX score as a measure of the severity of CAD assessed using the SYNTAX score using the application at https://syntaxscore.org/calculator/start.htm.

### 2.4 Statistical Analyses

The analysis was performed using SPSS version 19 (SPSS Inc., Chicago, IL, USA) and R3.6.3 software. Continuous variables were expressed as mean ± standard deviation (SD) and categorical variables as frequencies and percentages. For comparing the demographic and clinical characteristics between the STEMI and NSTEMI groups, independent *t* tests were used for continuous variables, and *χ* ^2^ tests or Fisher's exact tests were employed for categorical variables. Hardy‐Weinberg equilibrium for rs1801131 and rs1801133 polymorphisms was tested using the *χ* ^2^ test. Genotypic and allelic frequencies were compared between groups using *χ* ^2^ tests. The associations between rs1801131 and rs1801133 polymorphisms and the SYNTAX score were analyzed using one‐way ANOVA. To further explore the interaction effects between rs1801131 and rs1801133 polymorphisms on the SYNTAX score, two‐way ANOVA was performed. Post‐hoc analysis with Bonferroni correction was conducted to identify specific differences among genotypes. A two‐sided *p* ≤ 0.05 were considered statistically significant.

## 3 Results

### 3.1 Demographic Features of the Study Population

In present study included 65 males (67.7%) and 31 females (32.3%), and their mean age was 62.98 (±12.28) years. Furthermore, the mean SYNTAX score and the number of involved vessels were 15.65 (±10.03), and 3.03 (±0.75), respectively. There were no significant differences between several variables, including age, SYNTAX score, sex, HTN, IHD, HLP, renal stone, cerebrovascular accident (CVA), hypothyroidism, and PTE with STEMI and NSTEMI groups. The clinical characteristics of patients are presented in Table 1.

**Table 1:** Demographic characteristics of the participants in total population, STEMI, and NSTEMI groups.

Variable		Total population *N* = 96	STEMI *N* = 43	NSTEMI *N* = 53	*p*
Age (year)		62.98 ± 12.28	61.97 ± 12.73	63.81 ± 11.96	0.5^a^
SYNTAX score		15.65 ± 10.03	16.22 ± 8.98	15.2 ± 10.87	0.6^a^
Sex	Male	65 (67.7%)	32 (74.4%)	33 (62.3%)	0.2^b^
	Female	31 (32.3%)	11 (25.6%)	20 (37.7%)	
HTN	No	60 (63%)	27 (62.8%)	33 (62.3%)	0.99^b^
	Yes	36 (37%)	16 (37.2%)	20 (37.7%)	
IHD	No	75 (78.1%)	35 (81.4%)	40 (75.5%)	0.5^b^
	Yes	21 (21.9%)	8 (18.6%)	13 (24.5%)	
HLP	No	82 (85%)	37 (86%)	6 (14%)	0.9^b^
	Yes	14 (15%)	45 (84.9%)	8 (15.1%)	
renal stone	No	93 (96.9%)	42 (97.7%)	1 (2.3%)	*p* > 0.99^c^
	Yes	3 (3.1%)	51 (96.2%)	2 (3.8%)	
CVA	No	94 (97.9%)	42 (97.7%)	1 (2.3%)	*p* > 0.99^c^
	Yes	2 (2.1%)	52 (98.1%)	1 (1.9%)	
Hypothyroidism	No	94 (97.9%)	42 (97.7%)	1 (2.3%)	*p* > 0.99^c^
	Yes	2 (2.1%)	52 (98.1%)	1 (1.9%)	
PTE	No	95 (99%)	42 (97.7%)	1 (2.3%)	0.4^c^
	Yes	1 (1%)	53 (100%)	0	

*Note:* Data presented as mean ± standard deviation or number (%).

Abbreviations: CVA, cerebrovascular accident; HLP, hyperlipidemia; HTN, hypertension; IHD, ischemic heart disease; NSTEMI, Non‑ST‑Elevation Myocardial Infarction; PTE, pulmonary thromboembolism; STEMI, ST‑Elevation Myocardial Infarction; SYNTAX, Synergy between Percutaneous Coronary Intervention with Taxus and Cardiac Surgery.

a *t* test.

b *χ* ^2^ test.

^c^ Fisher exact test.

### 3.2 Genotype Distribution of Rs1801131 and Rs1801133 in STEMI and NSTEMI

Hardy–Weinberg equilibrium was maintained in the patients for rs1801131 and rs1801133, as shown in Table 2. The frequency of mutant alleles (rs1801131 and/or rs1801133) in the patients diagnosed with STEMI and NSTEMI in South Khorasan was 82.3% (Table 3). It means only 17.7% of participants have wild genotypes of studied SNPs.

**Table 2:** Hardy–Weinberg equilibrium for rs1801131 and rs1801133.

SNP	Genotype	Observed heterozygosity	Expected heterozygosity	*P* of Hardy Weinberg
Rs1801131	CC	0.14	0.15	0.99
	AC	0.48	0.47	
	AA	0.37	0.77	
Rs1801133	TT	0.06	0.07	0.99
	CT	0.39	0.38	
	CC	0.54	0.54	

**Table 3:** The frequency of rs1801131 and rs1801133 genotypes simultaneously.

Rs1801133/Rs1801131	Frequency *N* (%)
TT/CC	1 (1.0%)
TT/AC	1 (1.0%)
CT/CC	4 (4.2%)
TT/AA	4 (4.2%)
CC/CC	9 (9.4%)
CT/AC	19 (19.8%)
CT/AA	15 (19.8%)
CC/CC	26 (27.1%)
CC/AA	17 (17.7%)

The frequency of rs1801131 and rs1801133 genotypes in STEMI and NSTEMI patients are given in Table 4. The frequency of rs1801131 genotypes were 14.6% (CC), 47.9% (AC), and 37.5% (AA) in all patients, whereas the frequency of rs1801133 genotypes were 6.3% (TT), 39.6% (CT) and 54.2% (CC), respectively.

**Table 4:** The genotypic distribution for rs1801131 and rs1801133 in STEMI and NSTEMI patients.

SNP	Genotype	Total	STEMI *N* (%)	NSTEMI *N* (%)
Rs1801131	CC	14 (14.6%)	6 (42.9%)	8 (57.1%)
	AC	46 (47.9%)	22 (47.8%)	24 (52.2%)
	AA	36 (37.5%)	15 (41.7%)	21 (58.3%)
Rs1801133	TT	6 (6.3%)	3 (50.0%)	3 (50.0%)
	CT	38 (39.6%)	16 (42.1%)	22 (57.9%)
	CC	52 (54.2%)	24 (46.2%)	28 (53.8%)

### 3.3 Association Between rs1801131 and rs1801133 With Syntax Score in STEMI and NSTEMI Patients

**Figure 1:**
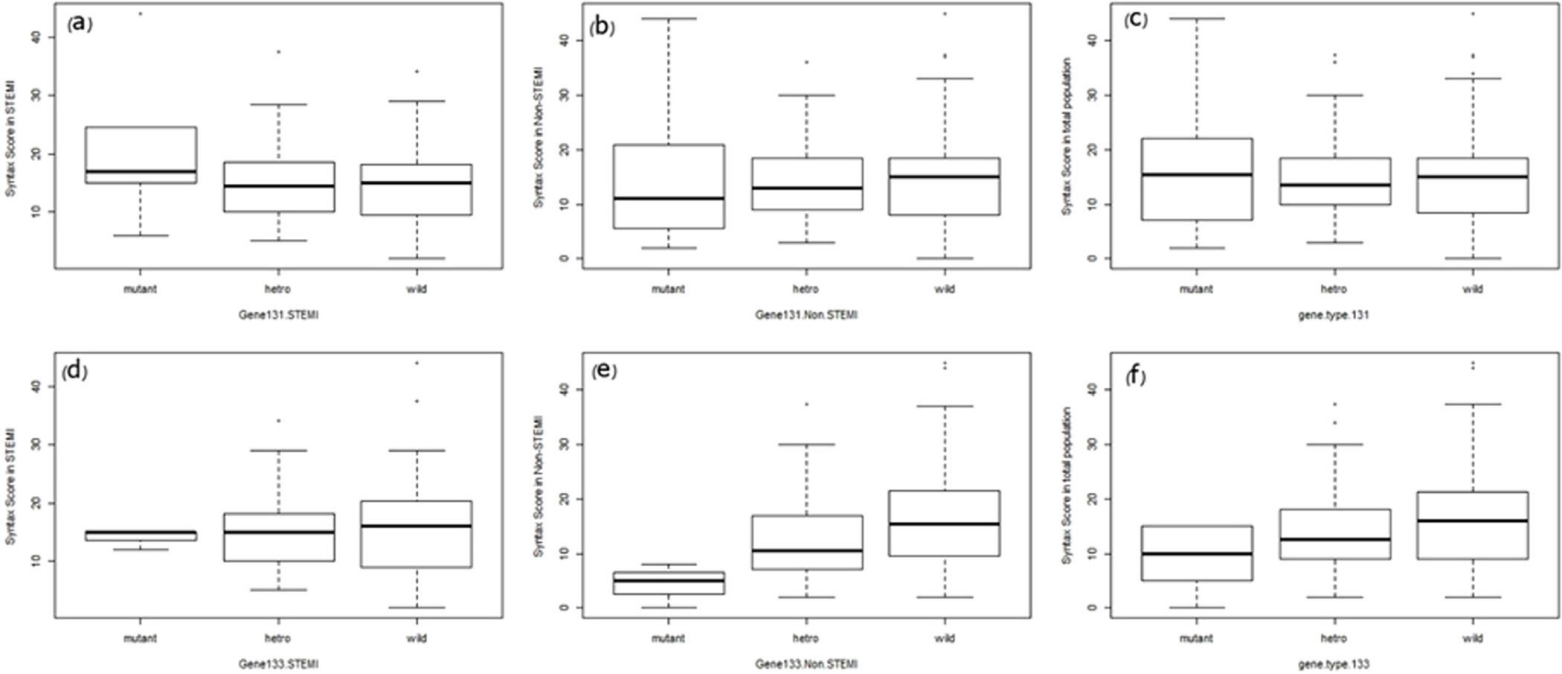
Boxplot of SYNTAX score by rs1801131 and rs1801133 in STEMI and NSTEMI and all participants (a) SYNTAX score in STEMI group with rs1801131, (b) SYNTAX score in NSTEMI group with rs1801131, (c) SYNTAX score in all participants with rs1801131, (d) SYNTAX score in STEMI group with rs1801133, (e) SYNTAX score in NSTEMI group with rs1801133, (f) SYNTAX score in all participants with rs1801133, #: significant difference with wild status in 0.1 level.

There are no significant differences between SYNTAX score in STEMI (*p* = 0.44), NSTEMI (*p* = 0.91), and all participants (*p* = 0.68) for rs1801131 (Figure 1). Furthermore, no association was found between SYNTAX score and rs1801133 in STEMI (*p* = 0.92), NSTEMI (*p* = 0.07), and all patients (*p* = 0.11).

Due to the simultaneous effects of genotypes on the SYNTAX score, the effect of each SNP in the presence of the other SNP on the SYNTAX score was calculated using the two‐way analysis of variance. Because the interaction effects of each SNP were not significantly correlated with the SYNTAX score, the main effects of the SNPs are interpretable in three models (Table 5). Two‐way analysis of variance and post‐hoc analysis indicated that rs1801133 could reduce the SYNTAX score in NSTEMI (*p* = 0.03) and total patients (*p* = 0.03).

**Table 5:** The results of the two‐way and post‐hoc test for investigating the association between rs1801131 and rs1801133 and SYNTAX score in various groups (total population, STEMI, and NSTEMI).

Model	SNPs	Genotype	SYNTAX score	*p*	Main effect comparison Post‐Hoc test
Total population	Rs1801133	TT	9.58 (4.93)	0.03	CT‐CC: *p* = 0.02 TT‐CC: *p* = 0.09
		CT	12.61 (1.99)		
		CC	18.43 (1.50)		
	Rs1801131	CC	17.46 (3.51)	0.79	—
		AC	14.85 (1.19)		
		AA	15.97 (1.85)		
STEMI	Rs1801133	TT	14.25 (5.72)	0.74	—
		CT	16.17 (2.35)		
		CC	17.27 (2.07)		
	Rs1801131	CC	18.35 (5.11)	0.53	—
		AC	15.08 (2.02)		
		AA	15.27 (2.80)		
NSTEMI	Rs1801133	TT	4.5 (6.5)	0.03	TT‐CC: *p* = 0.04
		CT	12.16 (2.44)		
		CC	19.14 (2.28)		
	Rs1801131	CC	15.12 (3.75)	0.96	—
		AC	11.63 (3.84)		
		AA	12.58 (2.99)		

## 4 Discussion

MTHFR plays a central role in homocysteine metabolism in homocysteine remethylation to methionine. MTHFR gene polymorphisms, such as rs1801131 and rs1801133, reduce the enzyme's efficiency in homocysteine remethylation, leading to elevated homocysteine levels. This elevation contributes to endothelial dysfunction, oxidative stress, and atherogenesis, which are key mechanisms in the progression and severity of CAD. By disrupting homocysteine metabolism, these variants have a direct impact on vascular health and lesion severity. The rs1801131 CC genotype decreases the enzyme activity of *MTHFR* by 40% more than the AA genotype, and the TT genotype has approximately 80% less activity of *MTHFR* compared with the CC genotype [15]. In rs1801133 cytosine mutates to thymine (677 C > T), which leads to replacing alanine with valine at the protein position A222V and the allelic position C677T, causing the downregulation of MTHFR [16, 29]. Considering the genetic diversity, there are various genes and phenotypes in different ethnicities because of the environmental and evolutionary variety [17]. Thus, it is crucial to study the correlation between genetic variations and the risk or severity of disease in various populations. Although over recent years, several studies have focused on the relationship between variants of the *MTHFR* gene and the susceptibility of CAD, there are not enough investigations on *MTHFR* polymorphisms and the severity of CAD. Hence, we aimed to study whether *MTHFR* common variants are correlated with the severity of coronary lesions in patients with STEMI and NSTEMI in the Iranian population.

Based on our results, the cumulative frequency of genotypes indicated that 82.3% of the population of South Khorasan have a mutation in rs1801131 and/or rs1801133 (Table 3). Hence, rs1801131 and/or rs1801133 might be useful biomarkers, especially in South Khorasan, Iran. Several studies have reported that the *MTHFR* polymorphisms are correlated with the susceptibility of CAD in various populations [14, 18, 19, 20, 29]. Research by Morita on 362 Japanese men diagnosed with CAD showed that this polymorphism in the *MTHFR* C677T position is higher in the case group than in controls [21]. More research on the Indian population reported the correlation between *MTHFR* polymorphisms and the susceptibility of CAD [22, 23]. A large number of studies on the Iranian population also indicated the same results [24, 25, 26, 27, 28]. Attar et al. reported that the C/T genotype in rs1801133 is an important factor in susceptibility to CAD in the Iranian population, particularly in females [28]. It is reported that the T allele of the rs1801133 can elevate the risk and progression of CAD [29]. The frequency of this allele is significantly higher in patients with CAD and may be correlated with CAD severity [15, 23], which was in line with a meta‐analysis conducted on the Chinese population [30]. These results have proposed that these polymorphisms can be presented as the potential genetic risk factors for CAD to predict the disease. However, there are some conflicting results [13]. Alroy et al. investigating the American population reported that the TT genotype of the C677T variant was not associated with CAD [31]. Another meta‐analysis showed that TT genotype versus CC in rs1801133 polymorphism could not increase the risk of CAD [32]. The conflicting results of this research may be due to various populations studied in population stratification or the small size of samples. Investigating the interactions of multiple effects of various genes, haplotypes, and the environment is required to study complicated diseases [33].

The main findings in this study were a significant association between rs1801133 with the effect of rs1801131 on SYNTAX score in NSTEMI (*p* = 0.03) and total patients (*p* = 0.03). As presented in Table 5, the first model demonstrated the simultaneous effects of rs1801131 and rs1801133 on SYNTAX score in the total population, which suggested that with the lack of rs1801131 effect, a significant difference in SYNTAX score emerges in various variants of rs1801133 (*p* = 0.032). This difference resulted in significant differences in the SYNTAX score between CC and CT (*p* = 0.02). The second model, indicating the simultaneous effects of rs1801131 and rs1801133 on SYNTAX score among STEMI patients, proposed that there is no correlation between these SNPs and the SYNTAX score. The third model presented the simultaneous effects of rs1801131 and rs1801133 among NSTEMI patients. It is observed that without the effect of rs1801131, there is a significant difference in SYNTAX score and rs1801133 variants (*p* = 0.032), which can be due to TT and CC variants of this SNP (*p* = 0.04). Therefore, regarding simultaneous effects among the genes in NSTEMI patients, a significant difference was observed between the SYNTAX score and the variants of rs1801133. Rs1801133 may affect the DNA methylation, leading to a change in the lipid metabolism, and be involved in CAD [29]. Thus, this SNP could be a valuable tool for predicting the severity of CAD. Our results were consistent with some research and disagreed with others. Bouzidi et al. reported no significant correlation between rs1801133 polymorphism in *MTHFR* in CAD patients with the high levels of homocysteine and the severity of CAD expressed by GS (*p* = 0.075) [14]. The lack of this association may be due to interactions between rs1801133 and other genetic risk factors. Moreover, the medications and nutritional intake of folate, vitamin B6, and B12 deficiency as co‐factors, hypertension, and smoking affect serum homocysteine and probably influence the concentration of homocysteine [34, 35].

*MTHFR* can induce higher concentrations of homocysteine through inactivation of the active dimer, adversely resulting in the binding of the flavin adenine dinucleotide [36], which affects the methylation pathway of homocysteine and *MTHFR* activity [37]. Hence, the 677TT polymorphism has been suggested to be related to vascular oxidative stress and endothelial dysfunction because of high concentrations of 5‐methyltetrahydrofolate. Emerging evidence has revealed that hyperhomocysteinemia associated with MTHFR plays a significant role in atherogenesis and endothelial dysfunction. The rs1801133 variant has a more pronounced effect on 5‐MTHF levels than on homocysteine levels [38]. Additionally, elevated homocysteine can raise high‐density lipoprotein cholesterol (HDL‐C) levels by inhibiting the synthesis of Apolipoprotein A‐I (ApoA‐I) and promoting the release of HDL‐C [39].

## 5 Limitations

This study has several limitations that should be acknowledged. First, the sample size was relatively small, comprising only 96 patients. This limited sample size may reduce the generalizability of our findings to broader populations. Second, the study was conducted in a single hospital in South Khorasan, Iran, which may introduce regional biases and limit the applicability of the results to other geographic and ethnic groups. Additionally, as a retrospective study, it is subject to inherent biases related to data collection and patient selection. The study also did not account for the potential influence of environmental factors and lifestyle variables such as diet, smoking, and medication use, which could affect homocysteine levels and the severity of CAD. Finally, while we focused on the rs1801131 and rs1801133 polymorphisms of the MTHFR gene, other genetic variants and gene‐gene interactions that might influence the severity of CAD were not explored. Further research with larger, more diverse populations and prospective study designs is needed to validate and expand upon our findings.

## 6 Conclusion

In conclusion, our study identifies a significant association between the MTHFR polymorphism rs1801133 and the severity of CAD, as measured by the SYNTAX score in NSTEMI patients and the overall cohort. Despite the high prevalence of mutant alleles, rs1801131 did not show a similar correlation. These findings highlight the potential of rs1801133 as a genetic biomarker for assessing CAD severity. Further research with larger and more diverse populations is essential to validate these results and enhance understanding of genetic influences on cardiovascular disease.

## Author Contributions

**Behnam Nazarzadeh:** conceptualization, data curation, formal analysis, investigation, methodology, validation, visualization, writing–original draft, resources, funding acquisition, supervision. **Saeedeh Sadat Ghazanfari:** investigation, data curation, resources, funding acquisition, supervision. **Farzaneh Karimi:** data curation, methodology, validation, visualization, writing–original draft, writing–review and editing. **Seyed Ali Moezibady:** validation, conceptualization, methodology, data curation. **Fatemeh Salmani:** visualization, validation, formal analysis, funding acquisition, software. **Kazem Dastjerdi:** writing–original draft, writing–review and editing, visualization, validation, supervision, project administration, funding acquisition, formal analysis, data curation, conceptualization. **Hamidreza Mohammadi:** writing–review and editing, software, formal analysis, methodology, project administration, writing–original draft.

## Ethics Statement

Approval was obtained from the ethics committee Birjand University of Medical sciences (number of ethical approvals: IR. BUMS. REC.1397.351). The procedures used in this study adhere to the tenets of the Declaration of Helsinki.

## Consent

All participants have signed an informed consent which includes the consent for publication.

## Conflicts of Interest

The authors declare no conflicts of interest.

## Transparency Statement

The lead author Kazem Dastjerdi affirms that this manuscript is an honest, accurate, and transparent account of the study being reported; that no important aspects of the study have been omitted; and that any discrepancies from the study as planned (and, if relevant, registered) have been explained.

## Data Availability

The datasets and/or analysed data will be open access and available from the corresponding author on reasonable request.
